# TiO_2_ Nanobelt@Co_9_S_8_ Composites as Promising Anode Materials for Lithium and Sodium Ion Batteries

**DOI:** 10.3390/nano7090252

**Published:** 2017-09-02

**Authors:** Yanli Zhou, Qian Zhu, Jian Tian, Fuyi Jiang

**Affiliations:** 1 School of Environmental and Material Engineering, Yantai University, Yantai 264005, China; zhouyanli@ytu.edu.cn; 2 Key Laboratory of Colloid and Interface Chemistry, Ministry of Education School of Chemistry and Chemical Engineering, Shandong University, Jinan 250100, China; 879391733@163.com; 3 School of Materials Science and Engineering, Shandong University of Science and Technology, Qingdao 266590, China

**Keywords:** TiO_2_ nanobelt@Co_9_S_8_, mixed phases, cycling stability, lithium ion batteries (LIBs), sodium ion batteries (SIBs)

## Abstract

TiO_2_ anodes have attracted great attention due to their good cycling stability for lithium ion batteries and sodium ion batteries (LIBs and SIBs). Unfortunately, the low specific capacity and poor conductivity limit their practical application. The mixed phase TiO_2_ nanobelt (anatase and TiO_2_-B) based Co_9_S_8_ composites have been synthesized via the solvothermal reaction and subsequent calcination. During the formation process of hierarchical composites, glucose between TiO_2_ nanobelts and Co_9_S_8_ serves as a linker to increase the nucleation and growth of sulfides on the surface of TiO_2_ nanobelts. As anode materials for LIBs and SIBs, the composites combine the advantages of TiO_2_ nanobelts with those of Co_9_S_8_ nanomaterials. The reversible specific capacity of TiO_2_ nanobelt@Co_9_S_8_ composites is up to 889 and 387 mAh·g^−1^ at 0.1 A·g^−1^ after 100 cycles, respectively. The cooperation of excellent cycling stability of TiO_2_ nanobelts and high capacities of Co_9_S_8_ nanoparticles leads to the good electrochemical performances of TiO_2_ nanobelt@Co_9_S_8_ composites.

## 1. Introduction

Lithium-ion batteries (LIBs), as one of the most important energy storage devices, have attracted extensive attention due to their advantages of high energy density and long cycle life [1,2]. However, the practical application of LIBs is still restricted especially in electrical devices and hybrid electric vehicles (HEV). Electrodes materials are key factors to affect the electrochemical performance for energy storages devices [3]. The commercial graphite anode for LIBs cannot meet the ever-increasing requirement owing to its low specific capacity (372 mAh·g^−1^) and the safety problems [4]. Therefore, more attention has been focused on designing new anode materials to replace graphite [5,6,7,8,9,10]. Among various transition metal oxides, Titanium dioxide (TiO_2_) has been considered as new anode materials for both LIBs and SIBs because of its low cost, environmental friendliness, high voltage platform, and long cycling stability [11,12,13,14,15]. 

One-dimensional (1D) TiO_2_ nanomaterials such as nanowires, nanotubes, and nanorods have been investigated as anodes, which effectively improved the ionic and electronic transport properties compared to TiO_2_ nanoparticles [16,17,18,19]. TiO_2_ nanobelt is one of the potential candidates among these materials in energy storage fields [20,21]. However, the intrinsic low theoretical capacity of TiO_2_ still limits its wide application. Thus, many effective methods have been adopted to increase its inherent low specific capacity. An efficient way is to hybridize TiO_2_ with another active material with high capacity. The composites synthesized by TiO_2_ and metal oxides or sulfides delivered excellent lithium storage performances [22,23,24]. For instance, the 3D electrode by assembling Fe_2_O_3_ hollow nanorods onto highly oriented TiO_2_ nanotube arrays delivered a high capacity of over 600 mAh·cm^−2^ at a current density of 100 mA·cm^−2^ after 50 cycles [23]. Furthermore, the TiO_2_@MoS_2_ hybrid exhibited a reversible capacity of 710 mAh·g^−1^ after 100 cycles at 100 mA·g^−1^ [24]. Another common method is to change the crystal phases of TiO_2_ to enhance its electrochemical performance. Very recently, TiO_2_-B anode materials have attracted great interest for both LIBs and SIBs [25,26,27,28]. They presented better electrochemical performances than the other phases of TiO_2_ due to their open tunnel structure and pseudocapacitive lithium storage properties [29,30]. Besides, it has been reported that the anatase/TiO_2_-B coherent interfaces could contribute to additional lithium storage, leading to better electrochemical performances than single phase TiO_2_ [31]. TiO_2_-B related composites have also become the research focus for LIBs. For example, the hierarchical TiO_2_-B nanowire@*α*-Fe_2_O_3_ composites exhibited better cycling stability than pure TiO_2_-B [32]. In addition, the TiO_2_-B nanoribbons anchored with NiO nanosheets as anode materials also displayed good cycling stability at a large rate of 5 C [33]. Cobalt sulfides as high capacity anodes have so many distinct advantages, such as the good conductivity, low electrode polarization and good thermal stability compared with transition metal oxides. However, to our knowledge, the composites of anatase/TiO_2_-B nanobelt and cobalt sulfides have not been reported yet for both LIBs and SIBs.

In this paper, TiO_2_ nanobelt@Co_9_S_8_ composites have been successfully obtained via a solvothermal reaction and high-temperature calcination process. The composites as anode materials for both LIBs and SIBs present good electrochemical performances, which is better than single TiO_2_ nanobelts and Co_9_S_8_ nanoparticles. The high reversible capacity, good cycling stability and rate capability of TiO_2_ nanobelt@Co_9_S_8_ composites are likely attributed to the synergistic effect of Co_9_S_8_ nanoparticles and anatase/TiO_2_-B nanobelts. 

## 2. Materials and Methods

### 2.1. Synthesis of mixed Phase TiO_2_ Nanobelt@Co_9_S_8_ Composites

Mixed phase TiO_2_ nanobelts were prepared by a previously reported method [34]. TiO_2_ nanobelt@Co_9_S_8_ composites were synthesized via the following preparation procedure: First, 30 mg TiO_2_ nanobelts glucose aqueous solution (25 mL, 0.05 M) was dispersed by ultrasonic treatment for 2 min. Then 1.5 mmol of cobalt acetate were added into this solution and stirred for another 20 min to form a homogeneous dispersion. After that, 4.5 mmol of thiourea in ethylene glycol (25 mL) was added into the above mixture. The obtained dispersion was transferred to a Teflon-lined stainless steel autoclave and then heated at 180 °C for 10 h. After it was cooled to room temperature, the precipitate was collected, washed with deionized water and absolute alcohol thoroughly, and dried at 60 °C for 12 h. Finally, the TiO_2_ nanobelt@Co_9_S_8_ composites were obtained through annealing the precipitate at 650 °C for 10 h under Ar/H_2_ (5%) atmosphere. Co_9_S_8_ nanoparticles were also prepared according to the methods reported in previous paper [35].

### 2.2. Sample Characterization

X-ray diffraction (XRD) patterns were carried out by a Bruker D8 advanced X-ray diffractometer using monochromatic Cu Kα radiation (λ = 1.5418 Å). Transmission electron microscope (TEM) images and high-resolution transmission electron microscopy (HRTEM) images were achieved on a high-resolution transmission electron microscope (JEOL-2100, Akishima, Tokyo, Japan). Scanning electron microscope (SEM) images, mapping images, and energy dispersive spectrometer (EDS) spectrum were taken from a field-emission scanning electron microscope (FEI Nova 450, Hillsboro, OR, USA). Raman spectra were obtained on a MicroRaman spectrometer using a laser of 532 nm as an excitation (LabRAM HR Evolution, Kyoto, Japan). Nitrogen sorption isotherm was examined on a Micromeritics ASAP2020HD88 gas sorptometer at 77.3 K (Micromeritics, Norcross, GA, USA).

### 2.3. Electrochemical Measurements

Electrochemical performances of various electrodes were evaluated by CR2032 coin cells. The working electrode was fabricated by coating a mixture of 70 wt % of active material, 20 wt % of acetylene black, and 10 wt % of binder CMC (carboxyl methyl cellulose) in deionized water on a clean copper foil. Then the obtained foil was dried in vacuum at 60 °C for 10 h. The resulting foil was roll-pressed and punched into discs with a diameter of 12 mm. The mass loading of active material is estimated to 1.0–1.5 mg·cm^−2^. The coin cells were assembled in an Ar-filled glovebox. For LIBs, the lithium foil was used as the counter electrode, Celgard 2400 microporous polypropylene membrane as the separator, and a solution of 1 M LiPF_6_ in ethylene carbonate (EC) and dimethyl carbonate (DMC) (1:1 *v*/*v*) as the organic electrolyte. For SIBs, the sodium foil was used as the counter electrode, glass fiber was used as the separator, and the electrolyte is a solution of 1 M NaClO_4_ in EC and diethyl carbonate (DEC) (1:1 *v*/*v*) containing of 2 wt % fluoroethylene carbonate (FEC). Galvanostatic charge-discharge curves were acquired in a range of 0.01 to 3 V on the battery cyclers (Land CT2001A, Wuhan, China). Electrochemical impedance spectra (EIS) were carried out on an electrochemical workstation (AUTOLAB PGSTAT302N, Herisau, Switzerland) over a frequency range of 100 kHz to 0.01 Hz. Cyclic voltammetry (CV) curves were measured on an electrochemical workstation (CHI660E, Shanghai, China) over 0.01 to 3 V at a scanning rate of 0.1 mV·s^−1^. All the electrochemical tests were performed at 25 °C.

## 3. Results and Discussion

**Figure 1:**
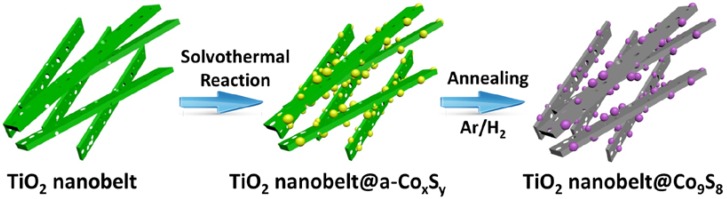
Preparation process of TiO_2_ nanobelt@Co_9_S_8_ composites.

Figure 1 shows the preparation process of TiO_2_ nanobelt@Co_9_S_8_ composites. Firstly, the mixed phase TiO_2_ nanobelts were prepared via a three step chemical reaction and annealing [34]. Secondly, the intermediate products labeled as TiO_2_ nanobelt@Co*_x_*S*_y_* were obtained by a solvothermal reaction, using mixed phase TiO_2_ nanobelts as growth templates and glucose as a linker. Finally, TiO_2_ nanobelt@Co_9_S_8_ composites were formed with the initial amorphous Co*_x_*S*_y_* changing into crystalline Co_9_S_8_ after an annealing treatment of 650 °C for 10 h under Ar/H_2_ atmosphere.

### 3.1. Characterization of Samples

**Figure 2:**
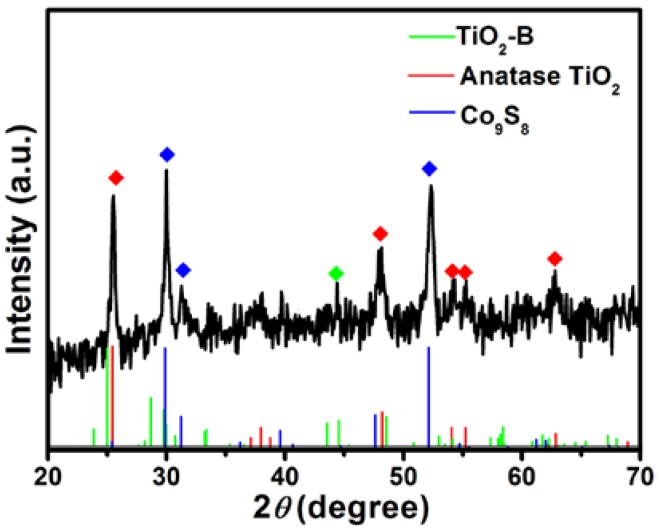
XRD pattern of the TiO_2_ nanobelt@Co_9_S_8_ composites.

Figure 2 shows the XRD pattern of obtained TiO_2_ nanobelt@Co_9_S_8_ composites. Three diffraction peaks marked with blue rhombus can be assigned to (311), (222) and (440) planes of cubic-phase Co_9_S_8_ (JCPDS Card, No. 65-1765) [8]. The diffraction peaks located at 2*θ*~25.3°, 48.0°, 53.9°, 55.1° and 62.7° can be identified as anatase TiO_2_ (JCPDS Card, No. 21-1272) [13]. The weak diffraction peak at 44.5° is attributed to TiO_2_-B (JCPDS Card, No. 46-1237) [16]. Meanwhile, mixed phase TiO_2_ nanobelt has been clearly demonstrated by the XRD patterns (Figure S1a and b). The Raman spectra of as-obtained TiO_2_ nanobelt and annealed treated TiO_2_ nanobelt also verify above results. As shown in Figure S1c and Figure S1d, two strong peaks located at 145 and 640 cm^−1^ are attributed to both anatase and TiO_2_-B, and one weak peak at 517 cm^−1^ is ascribed to anatase TiO_2_. All the remaining peaks are originated from TiO_2_-B [36]. EDS spectrum was measured to further verify the element composition and contents of the composites (Figure S2). The TiO_2_ contents (wt %) in the composites is estimated as ~64%, in which C and Al is from the conductive substrates, and the signal of Pt arises from the conductive coating of Pt on the sample surface by sputtering [37]. The EDS elemental mapping of composites also provides an even element distribution of Ti, Co and S (Figure S3). 

**Figure 3:**
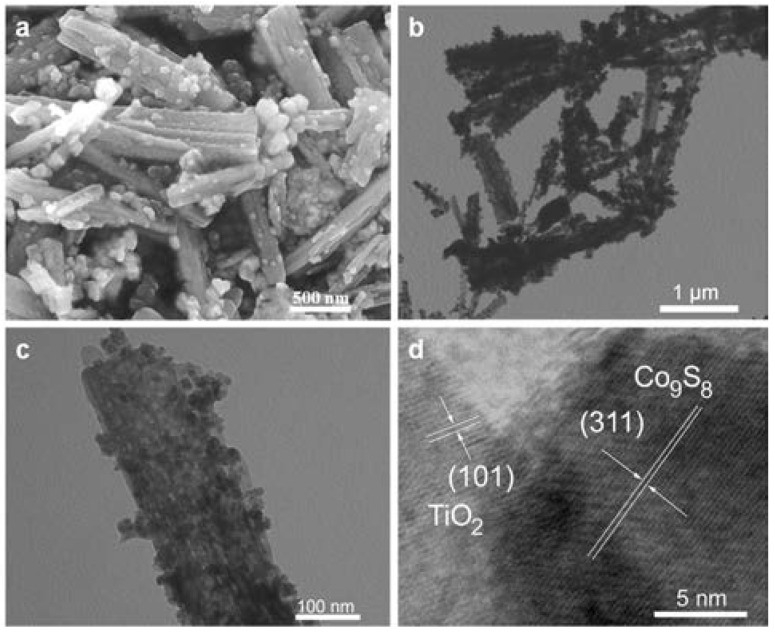
SEM image (**a**), TEM images (**b**,**c**) and HRTEM image (**d**) of the TiO_2_ nanobelt@Co_9_S_8_ composites.

The morphology of TiO_2_ nanobelt@Co_9_S_8_ composites was characterized by SEM and TEM techniques (Figure 3). The SEM image in Figure 3a clearly shows that some nanoparticles are evenly anchored on TiO_2_ nanobelts, implying the formation of TiO_2_ nanobelt@Co_9_S_8_ composites. Figure 3b exhibits its low magnified TEM images. It can be clearly observed that the TiO_2_ nanobelt is uniformly coated with some small nanoparticles, which agrees well with the SEM data. The high magnified TEM image in Figure 3c shows the clear morphology of single TiO_2_ nanobelt@Co_9_S_8_ composite. As can be seen, TiO_2_ nanobelt presents an irregular porous nanostructure after acidizing and annealing. The related HRTEM image is demonstrated in Figure 3d, the d-spacings at 0.299 and 0.34 nm agree well with those from (311) planes of Co_9_S_8_ and (101) planes of TiO_2_. The overlap of lattice fringes shows that small Co_9_S_8_ nanoparticles are grown on the surface of TiO_2_ nanobelt likely induced by some chemical force effect. The SEM and TEM images of TiO_2_ nanobelts and Co_9_S_8_ nanoparticles are also given as control samples (Figure S4). The nitrogen adsorption-desorption measurement was carried out to determine the surface area of TiO_2_ nanobelt@Co_9_S_8_ composites. As shown in Figure S5a, a type-IV isotherm with a distinct hysteretic loop for *P/P*_0_ ranges from 0.5 to 1.0 could be observed, suggesting the mesoporous structure in the product. The surface area of TiO_2_ nanobelt@Co_9_S_8_ composites is estimated as ~32.2 m^2^·g^−1^. The pore size ranges from 23 to 38 nm and the main peak is located at 38 nm (Figure S5b), which is in agreement with what observed in TEM images. As seen in the previous paper, the low surface area can suppress the unnecessary side reaction, such as inevitable electrolyte decomposition and formation of solid electrolyte interface (SEI) [38,39]. Thus, it could be supposed that the hierarchical mesoporous TiO_2_ nanobelt@Co_9_S_8_ composites as anode materials should present good lithium storage performance.

### 3.2. Electrochemical Performance of TiO_2_ Nanobelt@Co_9_S_8_ Composites for LIBs

**Figure 4:**
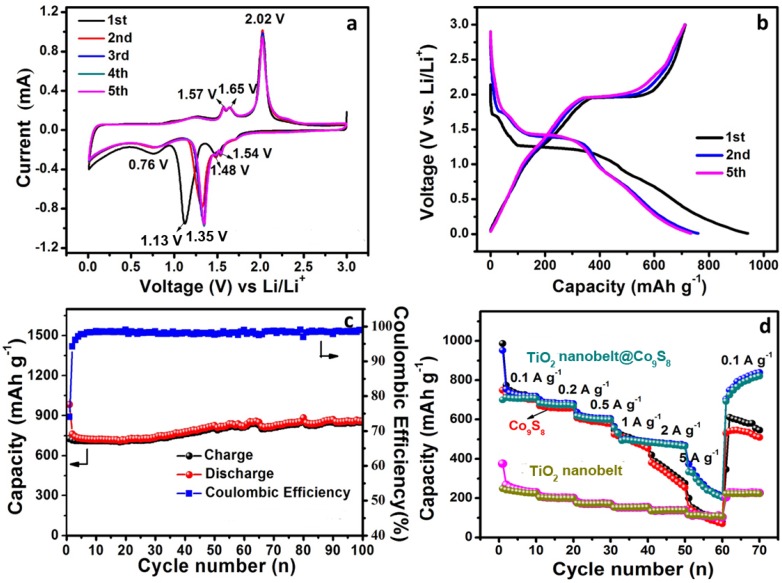
Cyclic voltammograms (**a**), galvanostatic discharge/charge profiles (**b**), cycling performances (**c**) of the TiO_2_ nanobelt@Co_9_S_8_ composites and (**d**) rate performances of the TiO_2_ nanobelt@Co_9_S_8_ composites, TiO_2_ nanobelts and Co_9_S_8_ nanoparticles.

The electrochemical performances of TiO_2_ nanobelt@Co_9_S_8_ composites for LIBs were tested (Figure 4). Figure 4a shows the cyclic voltammetry (CV) curves in a voltage window of 0.01–3.0 V at a scan rate of 0.1 mV·s^−1^. In the first cathodic scan, the strong cathodic peak located at 1.13 V is likely to come from the reduction of Co_9_S_8_ to metallic Co, which is ascribed to the conversion reaction: Co_9_S_8_ + 16Li^+^ → 9Co + 8Li_2_S [40]. A small wide peak located at~1.70 V is attributed to the formation of anatase Li*_x_*TiO_2_, corresponding to reaction equations: *x*Li^+^ + *x*e^−^ + TiO_2_ → Li*_x_*TiO_2_ [31]. The wide peak at 0.76 V is related to the electrolyte decomposition and formation of SEI layer [38,39]. Inversely, a sharp anodic peak around 2.02 V can be assigned to the reversible oxidation of metallic Co, which overlaps with the charge process of Li^+^ deintercalation from the anatase framework (Li*_x_*TiO_2_) [31]. Another two pairs of peaks located at 1.48/1.57 V and 1.54/1.65 V are ascribed to the surfaced-confined charge-transfer process (faradic pseudocapacitive lithium storage behavior) of TiO_2_-B [31]. The CV curves of TiO_2_ nanobelt further confirm the coexistence of anatase TiO_2_ and TiO_2_-B (Figure S6a). Figure 4b shows the galvanostatic discharge/charge voltage profiles of TiO_2_ nanobelt@Co_9_S_8_ composites for the first, second and fifth cycles over 0.01–3.0 V at 0.1 A·g^−1^. The first discharge curve shows multiple voltage plateaus mainly located at 1.6 and 1.2 V, which is in agreement with the redox peaks observed from CV results. The initial reversible capacity and coulombic efficiency of TiO_2_ nanobelt@Co_9_S_8_ composites could reach 714 mAh·g^−1^ and 74%, respectively. The irreversible capacity for the first cycle could result from the electrolyte decomposition and formation of SEI layer, which is very similar to that of transition metal oxides-based anodes [41,42]. Besides, it also arises from the solvated lithium intercalation and subsequent reduction of the solvent [43]. The irreversible capacities are largely dependent on the external surface area of the electrode and also plausibly related to the irreversible conversion reaction of Co_9_S_8_ for the first cycle and volume change of the electrode during the conversion process [44]. The cycling performance of TiO_2_ nanobelt@Co_9_S_8_ composites shows that a high reversible capacity of 889 mAh·g^−1^ can be achieved after 100 cycles (Figure 4c), which is far higher than that of single TiO_2_ nanobelt (Figure S6b). The capacity increase upon cycling is mainly from the pseudocapacitive lithium storage of TiO_2_ nanobelt and Co_9_S_8_ nanoparticles [8]. Such good electrochemical performance could be attributed to the designed hierarchical composites. The Co_9_S_8_ nanoparticles attached to TiO_2_ nanobelt can provide high capacity and improve conductivity for overall composites. Their small particle size and uniform dispersion of Co_9_S_8_ can effectively inhibit volume changes during cycling. Moreover, 1D TiO_2_ nanobelt can enhance the electron transfer efficiency, and the TiO_2_-B in the TiO_2_ nanobelt will lead to a higher reversible capacity compared to pure anatase TiO_2_ [45]. The interfaces between anatase and TiO_2_-B nanodomains can also contribute to additional lithium storage capacity [31]. More importantly, the anatase/TiO_2_-B nanobelt as backbone for Co_9_S_8_ can suppress the separation of Co_9_S_8_, its inherent cycling stability of TiO_2_ can also hinder the capacity loss of Co_9_S_8_ upon cycling. All the above factors facilitate the good electrochemical performances of TiO_2_ nanobelt@Co_9_S_8_ composites. The rate capability is another important kinetic factor to evaluate the electrochemical performance of TiO_2_ nanobelt@Co_9_S_8_ composites. As presented in Figure 4d, the average reversible capacity of TiO_2_ nanobelt@Co_9_S_8_ composites at a current density of 0.1, 0.2, 0.5, 1, 2 and 5 A·g^−1^ is 707, 676, 605, 502, 473 and 260 mAh·g^−1^, respectively, which is superior to those of the TiO_2_ nanobelt. The electrochemical performances of TiO_2_ nanobelt@Co_9_S_8_ composites at large current densities such as 1, 2 and 5 A·g^−1^ are superior to those of Co_9_S_8_ nanoparticles. Surprisingly, when the current density returns back to 0.1 A·g^−1^, the reversible capacity is still as high as 776 mAh·g^−1^, which is higher than initial capacity value. This phenomenon is likely attributed to the enhanced capacitance contribution resulting from the so-called electrochemical milling effect. All the results mentioned show that the TiO_2_ nanobelt@Co_9_S_8_ composites could be considered as potential anode materials to be applied for LIBs.

**Figure 5:**
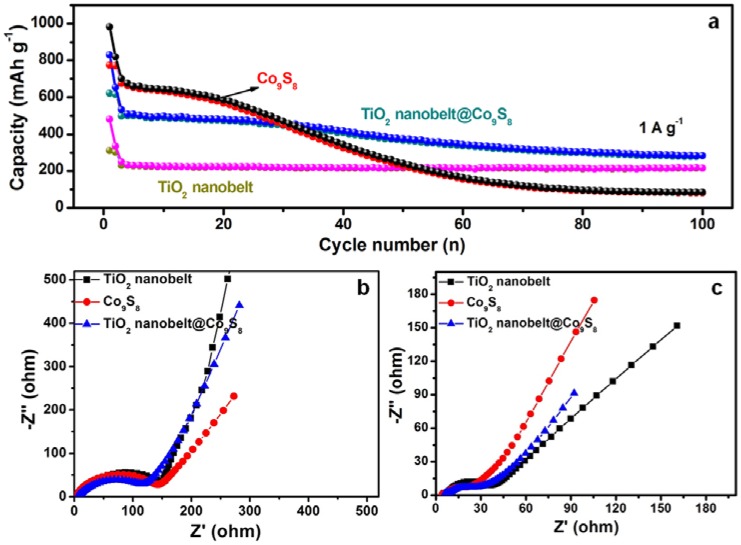
Cycling performances (**a**), electrochemical impedance spectra (EIS) of TiO_2_ nanobelts, Co_9_S_8_ nanoparticles and TiO_2_ nanobelt@Co_9_S_8_ composites: before cycling (**b**), and after cycling (**c**).

Figure 5a presents the cycling performances of TiO_2_ nanobelt@Co_9_S_8_ composites, TiO_2_ nanobelts and Co_9_S_8_ nanoparticles at 1 A·g^−1^. Although the cycling stability of TiO_2_ nanobelt is the best among three kinds of materials, its reversible capacity is only 216 mAh·g^−1^. The reversible capacity of TiO_2_ nanobelt@Co_9_S_8_ composites could retain at 369 mAh·g^−1^ after 100 cycles. The electrochemical impedance spectra (EIS) were tested to further investigate the electrode process kinetics of three kinds of electrodes (Figure 5b). For all the electrodes before cycling, the depressed semicircle at high-to-medium frequencies is attributed to charge-transfer impedance (*R*_ct_), and a slope at low frequencies is associated with ion diffusion process inside the electrode (constant phase element, CPE) [46]. After cycling, the depressed semicircles are related to two overlapped interface impedances (SEI and *R*_ct_) [35]. The phase angle of slope for TiO_2_ is close to 45°, suggesting a diffusion-controlled feature of lithium insertion/extraction. The phase angles of slope for both TiO_2_ nanobelt@Co_9_S_8_ composites and Co_9_S_8_ are greater than 45°, indicating significant capacitive component in lithium insertion/extraction [8]. Compared to the fresh electrodes, the decrease of *R*_ct_ for all the cycled electrodes suggests the electrochemically activation of anodes [47]. The relatively small *R*_s_, SEI and *R*_ct_ verify effective lithium and electron transfer of this composite during cycling.

### 3.3. Electrochemical Performance of TiO_2_ Nanobelt@Co_9_S_8_ Composites for SIBs

**Figure 6:**
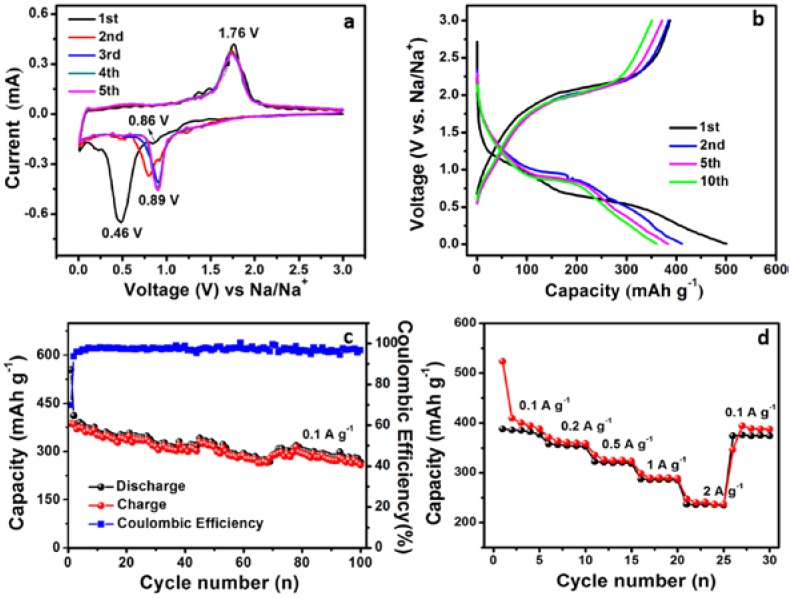
Cyclic voltammograms (**a**), galvanostatic discharge/charge profiles (**b**), cycling performances (**c**,**d**) rate performances of the TiO_2_ nanobelt@Co_9_S_8_ composites for SIBs.

The electrochemical performances of TiO_2_ nanobelt@Co_9_S_8_ composites, TiO_2_ nanobelt and Co_9_S_8_ nanoparticles for SIBs were also investigated (Figure 6). The CV curves are shown in Figure 6a, in the first cathodic scan, a strong peak located at ~0.46 V is likely to come from the formation of SEI film, and a small peak at 0.25 V is attributed to the intercalation process of Na^+^ into the TiO_2_ lattice [28]. The wide peaks located at ~1.13 V and 0.86 V are commonly attributed to the formation of Na_x_Co_9_S_8_ and further reduction process to Co and Na_2_S, respectively. The cathodic peak for the second cycle shifts to ~0.89 V, owing to irreversible structural rearrangement of Co_9_S_8_. For the anodic process, an intense oxidation peak at ~1.76 V could be assigned to the oxidation reaction of Co metal to form Co_9_S_8_ or Na*_x_*Co_9_S_8_ due to the irreversible reaction [48], which is in good accordance with previous reports [49]. The wide peak at ~0.52 V is associated with oxidation process of Na*_x_*TiO_2_ to TiO_2_ and Na_1−*x*_TiO_2_ [50]. The overlap of all the cathodic and anodic peaks in subsequent cycles demonstrates the good reversibility of TiO_2_ nanobelt@Co_9_S_8_ composites. Moreover, two sloping voltage plateau located at about 0.9 and 0.5 V appear in the first discharge curves, while for the following cycles, only one obvious discharge voltage plateau can be found, which is located at around 0.9 V. For all of the charge process, just a wide voltage plateau is observed. The wide voltage plateau indicate the overlap of electrochemical reaction of TiO_2_ and Co_9_S_8_ electrodes. All of the voltage plateaus in the discharge/charge curves of TiO_2_ nanobelt@Co_9_S_8_ composites can agree with the corresponding CV curves (Figure 6b).

Figure 6c shows the cycling performance of TiO_2_ naobelt@Co_9_S_8_ composites at 0.1 A·g^−1^. The initial discharge and charge capacity can reach 554 and 387 mAh·g^−1^, respectively. After 100 cycles, the reversible capacity can still maintain at 258 mAh·g^−1^, displaying good cycling stability of TiO_2_ naobelt@Co_9_S_8_ composites. The rate capability of TiO_2_ nanobelt@Co_9_S_8_ composites are shown in Figure 6d. The reversible capacity is 388, 358, 322, 288 and 237 mAh·g^−1^ at 0.1, 0.2, 0.5, 1 and 2 A·g^−1^, respectively. When the current density goes back to 0.1 A·g^−1^, the reversible capacity can return to 374 mAh·g^−1^. The cycling performance and rate capacity of TiO_2_ nanobelt@Co_9_S_8_ composites are better than those of single TiO_2_ and Co_9_S_8_ for SIBs (Figure S7). 

The EIS of three kinds of electrodes for SIBs were also measured (Figure S8), compared to fresh electrodes, all of the cycled electrodes exhibit smaller *R*_ct_, and the *R*_ct_ of TiO_2_@Co_9_S_8_ is located between TiO_2_ and Co_9_S_8_, which is possibly resulted from the good conductivity of Co_9_S_8_. The phase angles of slope for these electrodes are similar to those of LIBs. The TiO_2_ nanobelt@Co_9_S_8_ composites with good electrochemical performances are of great potential to be used for both LIBs and SIBs.

## 4. Conclusions

In summary, we have successfully synthesized mixed phase TiO_2_ nanobelt@Co_9_S_8_ composites. As new anode materials, they show high specific capacities and good cycling performances for both LIBs and SIBs. After 100 cycles, their reversible capacity for LIBs can retain at 889 and 369 mAh·g^−1^ at 0.1 and 1 A·g^−1^, respectively. Besides, an initial reversible capacity of 387 mAh·g^−1^ can be obtained at 0.1 A·g^−1^ for SIBs. Such good electrochemical performances of TiO_2_ nanobelt@Co_9_S_8_ composites could be attributed to the hierarchical nanostructure and synergistic effect of Co_9_S_8_ nanoparticles and anatase/TiO_2_-B nanobelts.

## Supplementary Materials

The following are available online at http://www.mdpi.com/2079-4991/7/9/252/s1, Figure S1: XRD patterns and Raman spectra of (a) and (c) as-prepared TiO_2_ nanobelts and (b) and (d) TiO_2_ nanobelts obtained by a heat treatment of 650 oC under Ar/H_2_ atmosphere, respectively, Figure S2: EDS spectrum of the as-prepared TiO_2_ nanobelt@Co9S8 composites (the inset is the molar ratio of element Ti, Co and S, respectively)., Figure S3: SEM image corresponding to EDS elemental mapping of Ti, Co and S of as-prepared TiO_2_ nanobelt@Co9S8 composites, Figure S4: SEM images (a) and (c), TEM images (b) and (d) of TiO_2_ nanobelts and Co9S8 nanoparticles, respectively, Figure S5: Nitrogen adsorption-desorption isotherm (a) and pore size distribution (b) of TiO_2_ nanobelt@Co9S8 composites at 77.3 K, Figure S6: CV curves (a) of TiO_2_ nanobelts for the first five cycles at a scan rate of 0.1 mV·s^−1^ and cycling performances (b) of TiO_2_ nanobelts at 0.1 A·g^−1^, Figure S7: Cycling performances (a) and (b) at 0.1 A·g^−1^, rate capacities (c) and (d) at different current densities of TiO_2_ nanobelts and Co9S8 nanoparticles for SIBs, Figure S8: Electrochemical impedance spectra (EIS) of (a) before cycling and (b) after cycling 30 cycles of TiO_2_ nanobelts, Co9S8 nanoparticles and TiO_2_ nanobelt@Co9S8 composites.
