# Supplementary material for: TiO2 Nanobelt@Co9S8 Composites as Promising Anode Materials for Lithium and Sodium Ion Batteries
Source: Nanomaterials (Basel). 2017 Sep 2;7(9):252. doi: 10.3390/nano7090252 (PMC5618363; doi:10.3390/nano7090252)
Supplement: Supplementary file 1 [file nanomaterials-07-00252-s001.zip › nanomaterials-220936-SI-proofreading-1.pdf]

Supporting information

TiO_2 Nanobelt@Co_9S_8 Composites as Promising Anode Materials for Lithium and Sodium Ion Batteries

Yanli Zhou[1], Qian Zhu[2], Jian Tian[3,*] and Fuyi Jiang[1,*]

1 School of Environmental and Material Engineering, Yantai University, Yantai 264005, China; zhouyanli@ytu.edu.cn (Y.Z.)

2 Key Laboratory of Colloid and Interface Chemistry, Ministry of Education School of Chemistry and Chemical Engineering, Shandong University, Jinan 250100, China; 879391733@163.com (Q.Z.)

3 School of Materials Science and Engineering, Shandong University of Science and Technology, Qingdao 266590, China

* Correspondence: fyjiang@ytu.edu.cn (F.J.); jiantian@sdust.edu.cn (J.T.); Tel: 0535-6706039 (F.J.); 0532-86057929 (J.T.); Fax: 0535-6706038 (F.J.); 0532-86057929 (J.T.)

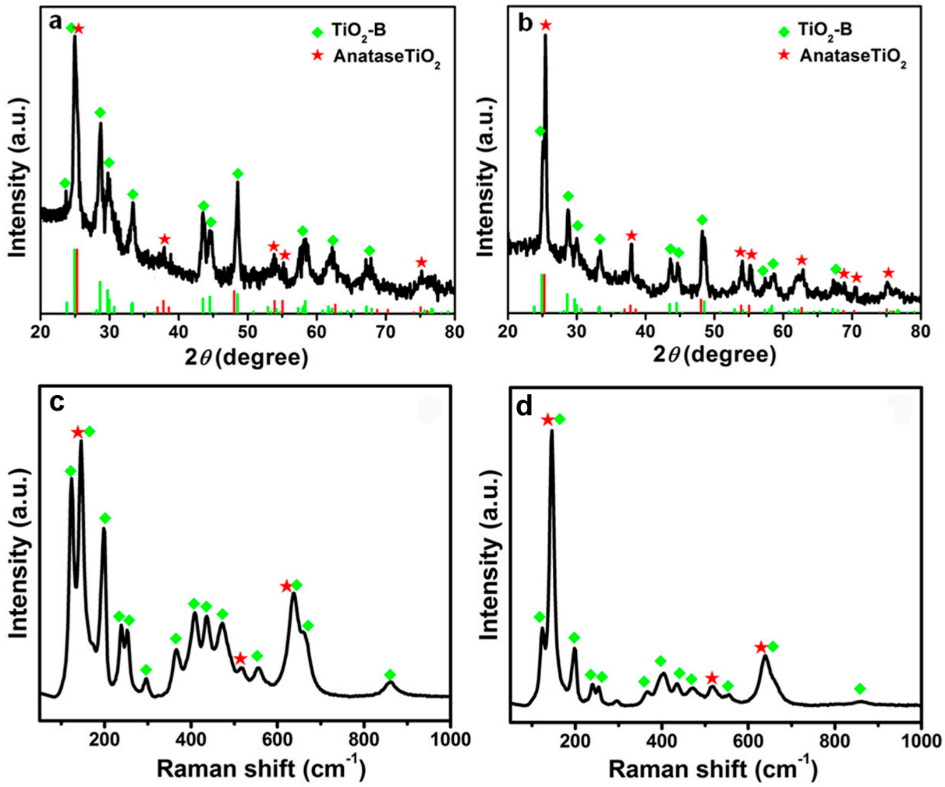

Figure S1. XRD patterns and Raman spectra of (**a**) and (**c**) as-prepared TiO_2 nanobelts and (**b**) and (**d**) TiO_2 nanobelts obtained by a heat treatment of 650 ^{o}C under Ar/H_2 atmosphere, respectively.

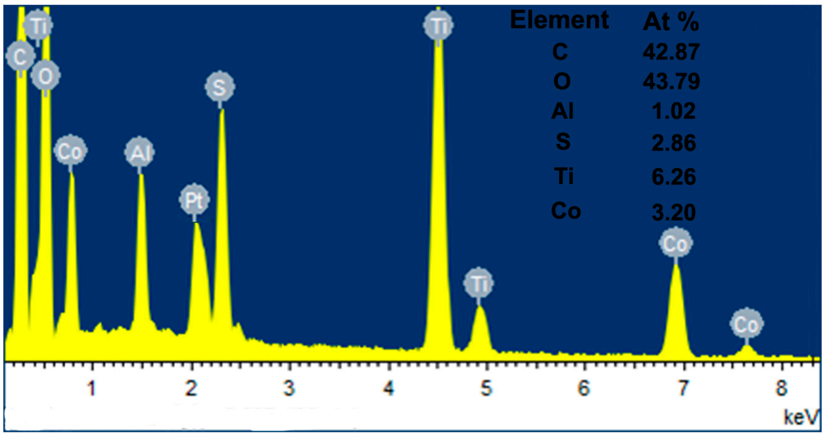

Figure S2. EDS spectrum of the as-prepared TiO_2 nanobelt@Co_9S_8 composites (the inset is the molar ratio of element Ti, Co and S, respectively).

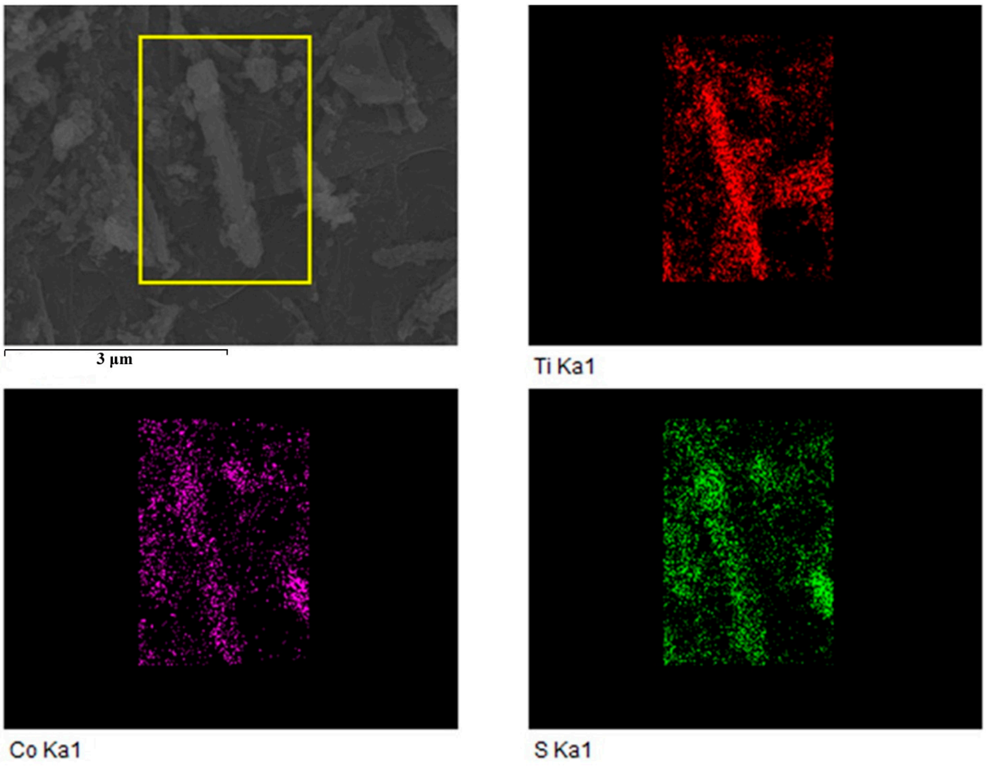

Figure S3. SEM image corresponding to EDS elemental mapping of Ti, Co and S of as-prepared TiO_2 nanobelt@Co_9S_8 composites.

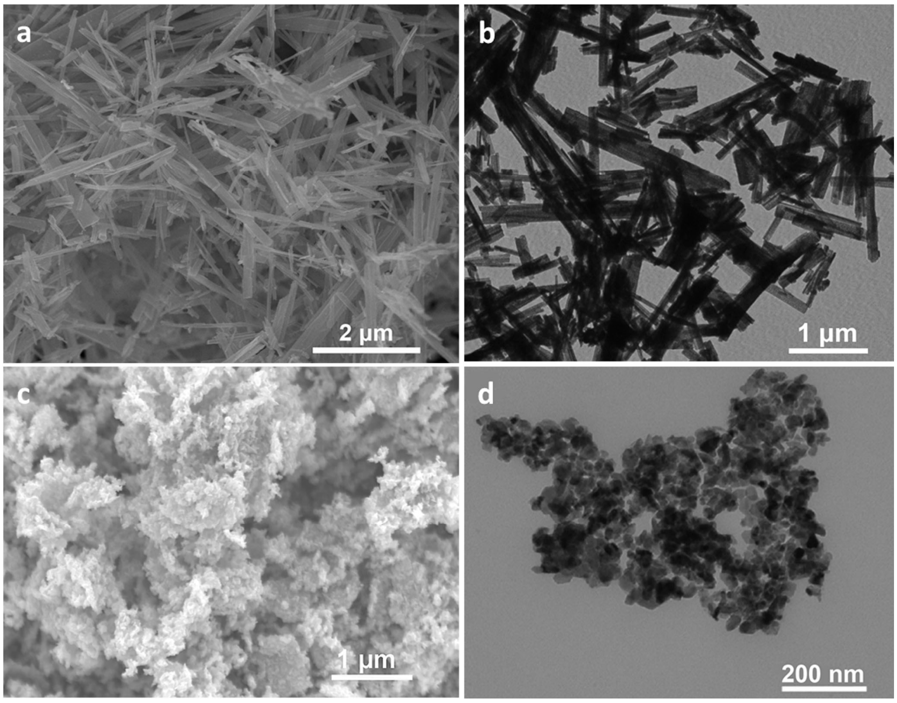

Figure S4. SEM images (**a**) and (**c**), TEM images (**b**) and (**d**) of TiO_2 nanobelts and Co_9S_8 nanoparticles, respectivley.

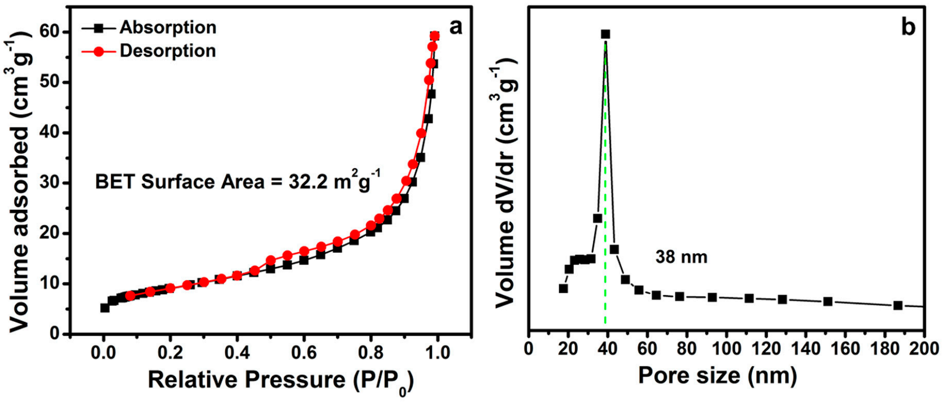

Figure S5. Nitrogen adsorption-desorption isotherm (**a**) and pore size distribution (**b**) of TiO_2 nanobelt@Co_9S_8 composites at 77.3 K.

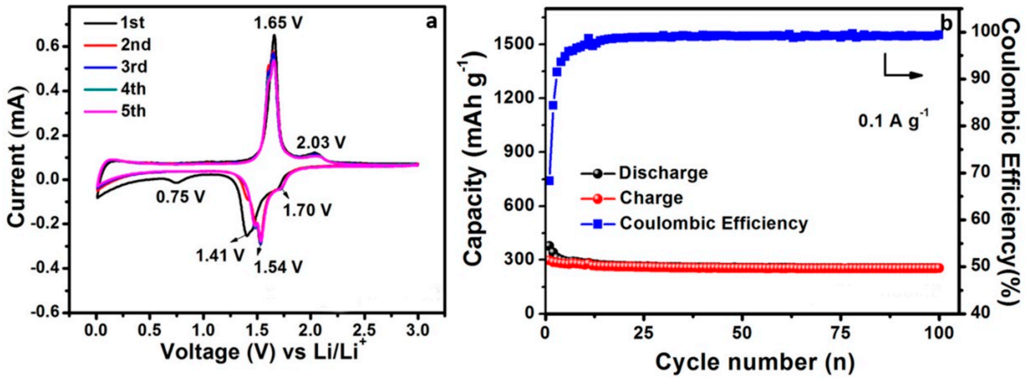

Figure S6. CV curves (**a**) of TiO_2 nanobelts for the first five cycles at a scan rate of 0.1 mV s^{-1} and cycling performances (**b**) of TiO_2 nanobelts at 0.1 $A{\cdot}g^{-1}$.

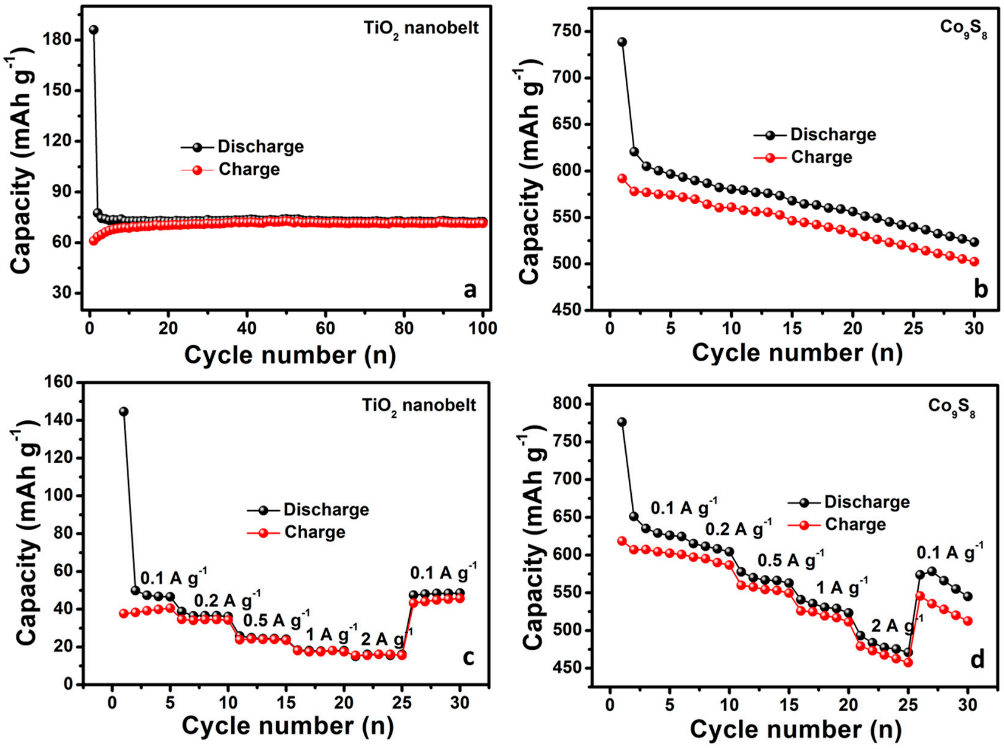

Figure S7. Cycling performances (**a**) and (**b**) at 0.1 $A{\cdot}g^{-1}$, rate capacities (**c**) and (**d**) at different current densities of TiO_2 nanobelts and Co_9S_8 nanoparticles for SIBs.

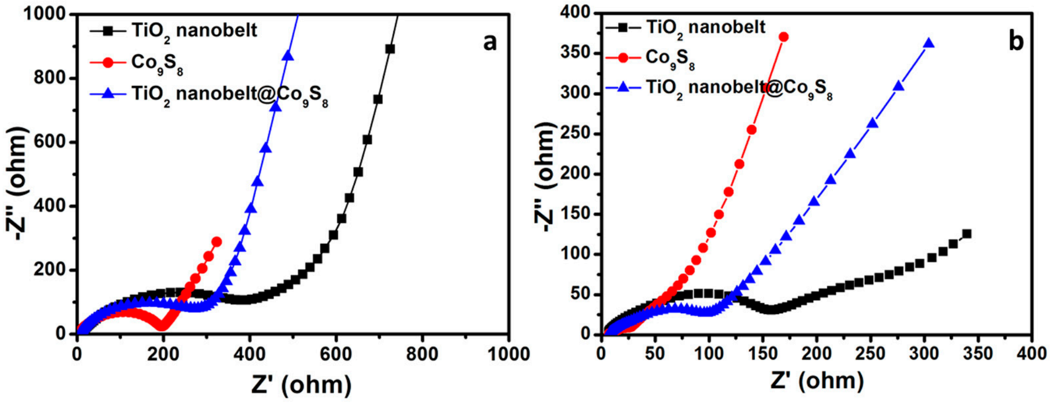

Figure S8. Electrochemical impedance spectra (EIS) of (**a**) before cycling and (**b**) after cycling 30 cycles of TiO_2 nanobelts, Co_9S_8 nanoparticles and TiO_2 nanobelt@Co_9S_8 composites.
