# Supplementary material for: TiO2 Nanobelt@Co9S8 Composites as Promising Anode Materials for Lithium and Sodium Ion Batteries
Source: Nanomaterials (Basel). 2017 Sep 2;7(9):252. doi: 10.3390/nano7090252 (PMC5618363; doi:10.3390/nano7090252)
Supplement: Supplementary file 1 [file nanomaterials-07-00252-s001.zip › nanomaterials-220936-SI-proofreading-1.doc]

**Supporting information**

TiO_2_ Nanobelt@Co_9_S_8_ Composites as Promising Anode Materials for Lithium and Sodium Ion Batteries

Yanli Zhou^1^, Qian Zhu^2^, Jian Tian^3,*^ and Fuyi Jiang^1,*^

^1^	School of Environmental and Material Engineering, Yantai University, Yantai 264005, China; zhouyanli@ytu.edu.cn (Y.Z.)

^2^	Key Laboratory of Colloid and Interface Chemistry, Ministry of Education School of Chemistry and Chemical Engineering, Shandong University, Jinan 250100, China; 879391733@163.com (Q.Z.)

^3^	School of Materials Science and Engineering, Shandong University of Science and Technology, Qingdao 266590, China

*	Correspondence: fyjiang@ytu.edu.cn (F.J.); jiantian@sdust.edu.cn (J.T.); Tel: 0535-6706039 (F.J.); 0532-86057929 (J.T.); Fax: 0535-6706038 (F.J.); 0532-86057929 (J.T.)

**Figure S1**. XRD patterns and Raman spectra of (**a**) and (**c**) as-prepared TiO_2_ nanobelts and (**b**) and (**d**) TiO_2_ nanobelts obtained by a heat treatment of 650 ^o^C under Ar/H_2_ atmosphere, respectively.

**Figure S2**. EDS spectrum of the as-prepared TiO_2_ nanobelt@Co_9_S_8_ composites (the inset is the molar ratio of element Ti, Co and S, respectively).

**Figure S3**. SEM image corresponding to EDS elemental mapping of Ti, Co and S of as-prepared TiO_2_ nanobelt@Co_9_S_8_ composites.

**Figure S4**. SEM images (**a**) and (**c**), TEM images (**b**) and (**d**) of TiO_2_ nanobelts and Co_9_S_8_ nanoparticles, respectivley.

**Figure S5**. Nitrogen adsorption-desorption isotherm (**a**) and pore size distribution (**b**) of TiO_2_ nanobelt@Co_9_S_8_ composites at 77.3 K.

**Figure S6**. CV curves (**a**) of TiO_2_ nanobelts for the first five cycles at a scan rate of 0.1 mV s^-1^ and cycling performances (**b**) of TiO_2_ nanobelts at 0.1 A·g^-1^.

**Figure S7.** Cycling performances (**a**) and (**b**) at 0.1 A·g^-1^, rate capacities (**c**) and (**d**) at different current densities of TiO_2_ nanobelts and Co_9_S_8_ nanoparticles for SIBs.

**Figure S8**. Electrochemical impedance spectra (EIS) of (**a**) before cycling and (**b**) after cycling 30 cycles of TiO_2_ nanobelts, Co_9_S_8_ nanoparticles and TiO_2_ nanobelt@Co_9_S_8_ composites.
